# Changing maternal, infant and young child nutrition practices through social and behaviour change interventions implemented at scale: Lessons learned from Alive & Thrive

**DOI:** 10.1111/mcn.13559

**Published:** 2023-09-21

**Authors:** Valerie L. Flax, Sujata Bose, Jessica Escobar‐DeMarco, Edward A. Frongillo

**Affiliations:** ^1^ RTI International Research Triangle Park North Carolina USA; ^2^ Alive & Thrive, FHI Solutions Washington District of Columbia USA; ^3^ Department of Public Health Sciences University of North Carolina Charlotte Charlotte North Carolina USA; ^4^ Arnold School of Public Health University of South Carolina Columbia South Carolina USA

**Keywords:** breastfeeding, complementary feeding, infant and young child feeding, maternal nutrition, social and behaviour change communication

## Abstract

Alive & Thrive (A&T) is an initiative designed to advance the implementation of maternal, infant and young child nutrition (MIYCN) social and behaviour change (SBC) at a large scale. The aims of this research were to: (1) describe A&T's SBC implementation processes and their impact based on a review of programme documents and peer‐reviewed publications and (2) gather lessons learned from key informant interviews (*N* = 23) with A&T staff and stakeholders in Bangladesh, Burkina Faso, Ethiopia, India, Nigeria and Vietnam. A&T's SBC approach used interpersonal communication, community mobilization and mass media to address knowledge gaps, strengthen self‐efficacy and shift social norms. The initiative used data for design and evaluation and facilitated scale and sustainability through close collaboration with governments and other stakeholders. A&T's approach increased exclusive breastfeeding, minimum meal frequency of children and use of iron and folic acid tablets by pregnant women, but had mixed impacts on early initiation of breastfeeding and maternal and child dietary diversity. Multiple SBC channels and frequent contacts strengthened the impact of SBC on MIYCN practices. Lessons learned included: using existing large‐scale platforms for interpersonal communication, improving counselling skills of health workers, delivering timely tailored messages, engaging key influencers to take specific actions, using research to address underlying behavioural concerns and maximize mass media reach and frequency, using simple memorable messages and employing additional channels to reach low media coverage areas. A&T developed and implemented at‐scale MIYCN SBC in multiple countries, providing lessons learned about intervention strategies, engagement of influencers and mass media campaign development, which governments and other implementers can adapt and replicate.

## Key messages

- A&T's at‐scale MIYCN SBC implementation model includes interpersonal communication, community mobilization, and mass media underpinned by strategic use of data and accompanied by advocacy.
- A&T's approach was effective for increasing exclusive breastfeeding, minimum meal frequency of children and use of iron and folic acid tablets by pregnant women, but impacts were mixed for other maternal and child nutrition indicators.
- The documentation of A&T's SBC implementation modalities across six countries and lessons learned from its implementation experiences can be used and adapted by governments and their development partners.

## Graphical abstract

Lessons learned from Alive & Thrive's at‐scale maternal, infant and young child nutrition social behaviour change model and implementation experiences across six countries can be used and adapted by governments and their development partners.

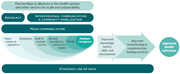

## 1 INTRODUCTION

Optimal maternal, infant and young child nutrition (MIYCN) practices are essential for maternal health, child survival and child growth and development (Black et al., 2013; Rollins et al., 2016). The World Health Organization (WHO) recommends that women have a minimum of eight health care contacts during antenatal care (ANC), receive counselling on healthy eating and staying physically active during pregnancy, and take daily oral iron and folic acid supplements (WHO, 2016). Other important maternal nutrition practices include pregnancy weight gain monitoring, starting ANC during the first trimester and taking calcium supplements. WHO and UNICEF recommend that children start breastfeeding within 1 h of delivery (early initiation), are exclusively breastfed until 6 months of age, receive a diverse diet and are fed a certain number of times per day based on their age and breastfeeding status (WHO, & UNICEF, 2021).

Recent data indicates major gaps in achieving recommended MIYCN practices in low‐income countries. UNICEF estimates that 48% of pregnant women aged 15 to 49 in low‐income countries during the period from 2015 to 2020 had a minimum of four ANC visits (UNICEF, 2021). Prevalence of iron and folic acid (IFA) supplementation compliance during pregnancy is not available globally; but in sub‐Saharan Africa, for example, compliance has been estimated at 39% (Fite et al., 2021). Among children in low‐income countries during the period from 2014 to 2020, UNICEF estimates that 57% started breastfeeding within 1 h of delivery, 53% were exclusively breastfed, 23% had minimum dietary diversity and 48% had minimum meal frequency (UNICEF, 2021).

Small‐scale programmes in low‐ and middle‐income countries (LMICs) have shown that social and behaviour change (SBC) interventions can be successful at addressing practice gaps for breastfeeding (Haroon et al., 2013; Imdad et al., 2011; Olufunlayo et al., 2019; Perez‐Escamilla et al., 2012; Sinha, et al., 2015), complementary feeding (Arikpo et al., 2018; Bhandari et al., 2004; Fabrizio et al., 2014; Guldan et al., 2020; Kilaru et al., 2005; Roy et al., 2005; Shi et al., 2009), ANC visits (Nair et al., 2016), and IFA supplementation (Gamboa et al., 2020; Ganjoo et al., 2022). Effective interventions have generally offered interpersonal communication, social support or both (Renfrew et al., 2012), with a few incorporating other elements such as mobile phone messaging (Downs et al., 2019; Flax et al., 2014; Patel et al., 2018). Interventions have been provided at health care facilities, in homes, or in communities, and have been offered by health care providers, various types of frontline health workers, and peers through individual or group counselling and support groups. SBC interventions have tended to focus on pregnant and lactating women, but a growing body of evidence points to the importance of involving key influencers, especially husbands and grandmothers, in interventions to support MIYCN practices (Martin et al., 2020, 2021).

Alive & Thrive (A&T) is a global initiative to save lives, prevent illness, and ensure healthy growth and development through improved breastfeeding, complementary feeding and maternal nutrition practices (https://www.aliveandthrive.org/en). Before A&T, studies showed that SBC interventions could be effective at changing MIYCN practices, but none of the interventions were implemented on a large scale (Arikpo et al., 2018; Bhandari et al., 2004; Fabrizio et al., 2014; Guldan et al., 2020; Haroon et al., 2013; Imdad et al., 2011; Kilaru et al., 2005; Olufunlayo et al., 2019; Perez‐Escamilla et al., 2012; Roy et al., 2005; Shi et al., 2009; Sinha et al., 2015). Implementing a programme at scale refers to taking a public health intervention or set of interventions known to be effective and expanding them to reach a large proportion of the target population while retaining effectiveness (Zamboni et al., 2019). The main goal of A&T is to implement SBC interventions at scale and generate evidence about scaled context‐specific models, effectiveness and implementation processes in diverse settings.

**Figure 1:**
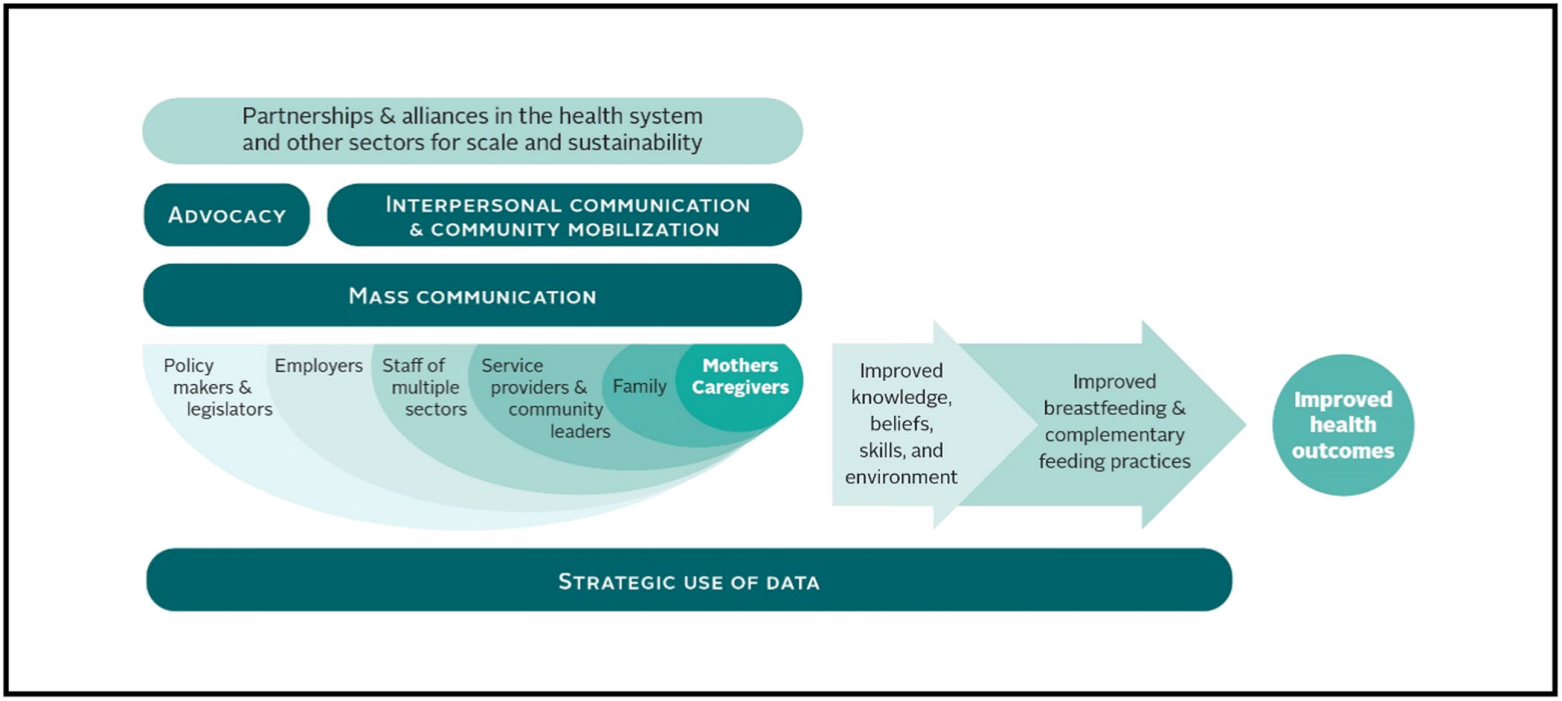
A&T programme conceptual model for improving IYCF practices.

A&T's first phase, from 2009 through 2014, was designed as a proof of concept that infant and young child feeding (IYCF) interventions could be delivered at scale and with impact in three countries (Bangladesh, Ethiopia and Vietnam), each representing different cultural, economic and health contexts. After its first phase of implementation, A&T developed an implementation framework for IYCF impact at scale (Figure 1), which is based on the socioecological model and serves as the foundation for assessment, planning, and implementation approaches for programme engagement in each country (Sanghvi et al., 2016). Beginning in 2014, A&T expanded to include India, Burkina Faso and Nigeria, applying the lessons learned from its first phase while continuing to generate lessons learned in Bangladesh, Ethiopia and Vietnam. Country‐specific activities continued in Bangladesh and Ethiopia. Continuity in Vietnam occurred via regional policy and advocacy efforts that included several Southeast Asia countries (Cambodia, Indonesia, Laos, Myanmar, Philippines, Thailand and Vietnam).

In May 2017, A&T was awarded a new 5‐year agreement, called Generation 2, which covered activities in Bangladesh and Burkina Faso and in Southeast Asia and West Africa, where A&T worked with stakeholders regionally to replicate strategies tested in initial countries within those regions. The Generation 2 award included support for further scale‐up of IYCF interventions and added a technical focus on maternal nutrition (Sanghvi, Nguyen, et al., 2022). Country‐specific awards continued being implemented in Ethiopia, India and Nigeria.

Although A&T SBC has been evaluated in individual countries, the initiative has not compiled lessons learned from its SBC activities across countries. Therefore, this paper documents learnings about A&T SBC design and implementation processes, the impact of A&T SBC on specific MIYCN practices based on completed evaluations, and experiences from A&T's implementation of SBC at scale that may be useful to other implementers within the broader nutrition community.

## 2 METHODS

This study focuses mainly on the interpersonal communication, community mobilization and mass media components of A&T's framework, which make up A&T's SBC approach, and touches on advocacy and strategic use of data as it relates to SBC design and implementation. The methods used were a desk review of A&T documents and key informant interviews (KIIs) with A&T staff and stakeholders in Bangladesh, Burkina Faso, Ethiopia, India, Nigeria and Vietnam.

### 2.1 Desk review

The desk review involved reading A&T programme documents, evaluation reports and peer‐reviewed publications to extract information about SBC intervention design, implementation, and impacts on IYCF and maternal nutrition practices. Programme documents selected for review included global and country SBC strategies, SBC implementation guides, annual reports, and SBC print and mass media materials, which provided information about A&T's SBC intervention design and implementation. The evaluation reports and peer‐reviewed papers were used to compile information about A&T's impacts on MIYCN practices. The extracted information was compiled in tables and summarized in text.

### 2.2 Key informant interviews

A total of 23 KIIs were conducted with A&T staff and stakeholders across the six target countries and the A&T Washington and regional offices. The number of KIIs by location were as follows: Bangladesh (3), Burkina Faso (3), Ethiopia (4), India (3), Nigeria (4), Vietnam (4) and Washington/Regional offices (2). Participants were selected for interviews from lists of A&T staff in each country and through consultation with A&T country directors about which staff and government counterparts would be most knowledgeable about A&T SBC design, implementation and lessons learned. The types of A&T staff included were SBC advisors, technical advisors and specialists, strategy advisors, monitoring and evaluation specialists, and country and regional directors. A&T staff who had been with the project since inception were prioritized for inclusion. The majority of those interviewed were current A&T staff, but a few former A&T staff or consultants were included.

The interviews and data analysis were conducted by one author who was external to A&T and was commissioned to conduct the learning assessment. This strategy eliminated the challenges of interrater reliability in coding and prevented conflicts of interest for two of the coauthors who were A&T staff at the time of data collection and analysis.

The KIIs were conducted on Zoom and digitally recorded. They were carried out in English, except in Burkina Faso where they were conducted in French. Interviews lasted approximately 60 to 90 min and were guided by a qualitative question guide, which included questions about lessons learned and challenges A&T faced related to interpersonal communication, community mobilization and mass media for breastfeeding, complementary feeding and maternal nutrition.

Recordings of the interviews were transcribed verbatim and analysed using thematic content analysis methods (Hsieh & Shannon, 2005). The same researcher who conducted the interviews read the transcripts several times and coded them by adding comments to sections of text in Microsoft Word. A data matrix was then created in Excel with themes and subthemes in rows and countries/locations in columns. Quotations and summaries of key themes were inserted into the matrix to facilitate analysis of SBC lessons learned across countries and selection of illustrative quotations. All coauthors had an opportunity to give input on the coding and analysis.

## 3 RESULTS

### 3.1 A&T SBC approach

Details of A&T's specific SBC activities carried out through implementing partners in each country are shown in Table 1. Interpersonal communication formed the bedrock of A&T's SBC activities. Across countries, A&T collaborated with governments, major community‐based implementers (e.g., BRAC in Bangladesh) or both to support the provision of face‐to‐face interpersonal communication between mothers and facility‐based health workers or community‐based health workers/volunteers on a large scale. Interpersonal communication was offered individually and in groups and was provided at health facilities, in communities or at women's homes, depending on the setting.

**Table 1:** A&T's IYCF and maternal nutrition SBC approaches by country.

Country	Channel	Activities
*Infant and young child feeding*		
Bangladesh	Interpersonal communication	■ Home visits: Monthly visits by community health volunteers, including cooking demonstrations ■ Post‐natal care visits: 12 visits from IYCF promoters ■ Antenatal care sessions: once monthly in a community member's home, led by community health workers ■ Health forums: Once monthly, attended by women and family members, led by community health workers ■ Mothers could call IYCF promoters for help with IYCF problems
	Community mobilization	■ Orientations with stakeholders ■ Sessions for religious leaders, fathers and male elders, government health officers, schoolteachers, informal health care providers, influential community members, adolescents, Maternal, Neonatal, and Child Health (MNCH) committee members ■ Video shows in media‐dark areas ■ Village theatre ■ Advocacy seminars for medical doctors
	Mass media and other campaign materials	■ Seven TV spots ■ Seven radio spots ■ Poster for doctors and health workers ■ Two job aids for frontline workers: IYCF, hand washing ■ Hand washing sticker for mothers and family members ■ Mobile phone counselling sticker ■ Newspaper inserts ■ Child feeding bowls
Burkina Faso	Interpersonal communication	■ Antenatal and post‐natal care visits: 8 contacts with government health workers ■ Women's group discussions at health facilities ■ Home visits by community health volunteers
	Community mobilization	■ Community events ■ Group discussions: Once per month, led by community workers
	Mass media and other campaign materials	■ Radio campaign (not aired through radio but aired during community events in impact evaluation intervention areas) ■ Counselling cards for health workers ■ Posters and mini‐posters for health facilities ■ Take‐home leaflets for mothers ■ Billboards
Ethiopia	Interpersonal communication	■ Home visits by health extension workers (HEWs) every 2 months and women's development army (WDA) every 2 weeks ■ Community group demonstrations and dialogues ■ Contacts with agricultural extension workers and development agents (Phase 2)
	Community mobilization	■ Model kebele committees and launch workshops ■ Target setting for Smart and Strong certificates ■ Group presentations/community conversations, including a session for fathers and cooking demonstrations ■ Sermons by priests about child feeding during fasting periods
	Mass media and other campaign materials	■ Seven TV spots, short dramatic film, video for leader mobilization, music video, instructional videos of food preparation ■ Seven radio spots, radio magazine programme, radio drama ■ Mobile video van/theatre shows ■ IYCF counselling cards for HEWs and WDAs ■ Job aids for HEWs and WDAs ■ Seven excellent actions card for households ■ Child nutrition card
India		■ Collaborated with the technical support units in Uttar Pradesh and Bihar to develop and/or test MIYCN SBC strategies for use as part of the Integrated Child Development Scheme ■ Worked with two organizations to integrate IYCF messaging into self‐help groups ■ Collaborated with government and development partners to create a national MIYCN SBC strategy and mass media plan
Nigeria	Interpersonal communication	■ Group and individual counselling by public and private health providers and traditional birth attendants ■ Home visits by community volunteers ■ Community meetings
	Community mobilization	■ Support group meetings for pregnant women/mothers ■ Religious meetings and sermons ■ Special events for World Breastfeeding Week and MNCH Week ■ Meetings of community‐based organizations for fathers
	Mass media and other campaign materials	■ Three TV spots ■ Three radio spots ■ TV and radio call‐in programmes ■ Text and WhatsApp messages to women/mothers ■ WhatsApp breastfeeding support group ■ Text messages to fathers ■ Sermon guides ■ Posters ■ Leaflets ■ Foldable pocket cards ■ Billboards ■ Vehicle wraps ■ Child feeding bowls
Vietnam	Interpersonal communication	■ Social franchise model at health facilities for individual and group counselling: 6–10 individual counselling contacts and 3–5 group sessions ■ Breastfeeding and complementary feeding support groups for women (remote areas)
	Community mobilization	■ Special events to encourage the use of franchise services ■ Community support groups for fathers and other caregivers of young children (remote areas)
	Mass media and other campaign materials	■ Five TV spots ■ Educational video ■ Job aids for health workers ■ Eight posters ■ Leaflets ■ Client booklets ■ Client brochure on services offered ■ Four invitation cards to counselling sessions ■ Bus wraps ■ Billboards ■ Face cloths and infant caps
*Maternal nutrition*		
Bangladesh	Interpersonal communication	■ Home visits: Visits by community health workers (once in the first trimester and monthly thereafter, assist with delivery) and community health volunteers (twice per month during pregnancy)
	Community mobilization	■ Husbands' forums: Husbands attended two forums during pregnancy, led by community health workers ■ Orientations for stakeholders, such as health care providers, village doctors, religious and local leaders, teachers ■ Orientations for government officials, social influencers and union council members
	Mass media and other campaign materials	■ Two video clips for women, their families, social influencers and local leaders ■ Audio for husbands' forums ■ Flipchart for husbands' forums ■ Two posters for government health facilities ■ Newspaper inserts ■ Community events and popular theatre
Burkina Faso	Interpersonal communication	■ Counselling by nurse‐midwives during antenatal care visits ■ Home visits
	Community mobilization	■ Meetings with women's groups, led by community mobilization agents and government health workers ■ Orientations for community leaders ■ Gatherings for grandmothers, husbands and other key influencers
	Campaign materials	■ Counselling flipchart for health workers ■ Job aid for health workers ■ Four posters ■ Leaflets for husbands, mothers‐in‐law
Ethiopia	Interpersonal communication	■ Counselling at health centres and health posts ■ Home visits by HEWs, including engaging husbands
	Community mobilization	■ Pregnant women conferences/mother support groups led by HEWs ■ Administrative ward (Kebele) and community meetings led by HEWs, WDAs, imams and community volunteers
	Campaign materials	■ Job aids for health workers ■ Posters ■ Maternal nutrition follow‐up card
India	Interpersonal communication	■ Facility‐based antenatal care sessions ■ Home visits: At least four visits by accredited social health activists (ASHAs), including engaging family members ■ Village health and nutrition days ■ Rural maternal and child care centre (Anganwadi) activities ■ Community‐based events
	Community mobilization	■ Husbands' forums led by ASHAs and a community lead ■ Community sensitization sessions ■ Sensitization sessions with doctors in the public system

Abbreviations: Anganwadi, rural maternal and child care centre in India; ASHA, accredited social health activist; HEW, health extension worker; IYCF, infant and young child feeding; Kebele, small administrative unit in Ethiopia; MIYCN, maternal, infant, and young child nutrition; MNCH, maternal, neonatal, and child health; SBC, social and behaviour change; WDA, women's development army.

The community mobilization component of A&T's SBC approach was intended to reach a broader segment of the population, shift MIYCN social norms and support interpersonal communication. This involved training or orienting different types of leaders, encouraging leaders to share messages using their platforms and conducting group activities for mothers and key influencers, especially fathers/husbands and grandmothers/mothers‐in‐law.

A&T used mass media as part of its IYCF SBC approach to reach scale, create awareness, shift social norms and contribute to behaviour change among the broader population and specific audience segments. The IYCF mass media strategies varied the most across countries (Sanghvi, Homan, et al., 2022), with the choice of media and target audiences selected based on formative research and media habits studies. Mass media using TV or radio was not included in A&T maternal nutrition programming as it operated by integrating contextualized packages of maternal nutrition interventions into ANC services and engaging mothers and key influencers through outreach from ANC providers. A&T developed a variety of other IYCF SBC materials (e.g., counselling cards, posters, leaflets, billboards, bus wraps, sermon guides, wall charts, social media materials, child feeding bowls) that were used to support interpersonal communication or community mobilization and to reinforce mass media. Printed campaign materials were included in the maternal nutrition interventions in most countries.

As compared to other countries, A&T employed a different strategy in India, where the main implementing partner provided technical assistance directly to the government and its partners by supporting and strengthening the existing government reproductive, maternal, newborn and child health (RMNCH) services.

### 3.2 Impact of A&T SBC interventions on IYCF and maternal nutrition practices

A&T collaborated with external researchers to carry out evaluations and implementation research to measure intervention impacts on IYCF and maternal nutrition practices. The study designs, impact findings and effect sizes are summarized in Table 2.

**Table 2:** Impact of A&T SBC on IYCF and maternal nutrition practices.

Country and years	References	Study design	Results
*Infant and young child feeding*			
Bangladesh, 2010–2014	Menon, Nguyen, Saha, Khaled, Kennedy, et al. (2016)	Cluster‐randomized impact evaluation with groups assigned to intensive or nonintensive interventions	*Breastfeeding:* Early initiation of breastfeeding (EIBF) + 16.7 percentage points (pp) Feeding of pre‐lacteals in first 3 days −49.3 pp Exclusive breastfeeding (EBF) + 36.2 pp
	Menon, Nguyen, Saha, Khaled, Sanghvi, et al. (2016)	Same as above	*Complementary feeding:* Minimum dietary diversity (MDD) + 16.3 pp Minimum meal frequency (MMF) + 14.7 pp Minimum acceptable diet (MAD) + 22.0 pp Consumption of iron‐rich foods +24.6 pp *Association of exposures with outcomes:* Interpersonal communication (IPC) alone with MMF and MAD IPC + mass media (MM) with MDD, MMF and MAD IPC + MM + community mobilization (CM) with MDD, MMF and MAD
	Kim et al. (2020)	Same as above	*Association of exposures with outcomes:* 4–6 visits in the last 6 months with EBF 1–3 visits in the last 6 months with MMF >6 visits in the last 6 months with MMF
Burkina Faso, 2015–2017	Cresswell et al. (2019)	Cluster‐randomized impact evaluation with groups assigned to intervention or control	*Breastfeeding:* EIBF 22.7% risk difference EBF 38.9% risk difference Received colostrum 20.0% risk difference Received no prelacteals 8.8% risk difference
Ethiopia, 2010–2014	Kim et al. (2016)	Pre/post‐intervention adequacy evaluation	*Breastfeeding:* EIBF + 13.7 pp EBF + 9.4 pp *Complementary feeding:* Introduction of solid, semisolid or soft foods +22.2 pp Consumption of iron‐rich foods +2.7 pp Consumption of grains +7.1 pp; legumes +13.8 pp; flesh foods +1.8 pp; eggs +7.8 pp; and vitamin A rich fruits and vegetables +6.5 pp *Association of exposures with outcomes:* Number of health post visits with EIBF Number of channels with EIBF, MDD and MAD Number of home visits in last 6 months with MDD, MMF and MAD Number of messages on community nutrition card recalled with MDD, MMF and MAD Number of radio spots heard with MDD and MAD
Ethiopia, 2015–2017	Kim et al. (2019)	Cluster‐randomized impact evaluation with groups assigned to intensive or nonintensive interventions; intensive intervention included an agricultural component	*Complementary feeding:* MDD + 6.6 pp MMF + 5.7 pp (not statistically significant) MAD + 5.5 pp Consumption of vitamin A rich fruits and vegetables +9.0 pp *Association of exposures with outcomes:* All exposures except A&T radio programme with MDD Hearing complementary feeding messages, attending community conversations, hearing A&T radio programme with MMF
Nigeria, 2016–2020	Flax, Fagbemi, et al. (2022)	Cluster‐randomized impact evaluation with groups assigned to intervention or control; analysis performed to measure impact from baseline to endline due to spillovers	*Breastfeeding:* EBF + 11 pp (difference‐in‐differences estimate, Kaduna) Baseline‐endline increases in the intervention arm: EIBF + 12 pp (Kaduna) EBF + 17 pp (Kaduna); +9 pp (Lagos) *Complementary feeding:* Baseline‐endline increases in the intervention arm: MDD + 20 pp (Kaduna); +19 pp (Lagos) *Association of exposures with outcomes:* IPC, CM and MM with EIBF, EBF and MDD in Kaduna IPC with EIBF, EBF and MDD in Lagos
Nigeria, 2018–2020	Flax, Ipadeola, Schnefke, Ralph‐Opara, et al. (2022)	Cohort study of private health facilities purposefully assigned to intervention or control; mothers enroled in the third trimester and followed up at 6 and 24 weeks post‐partum	*Breastfeeding:* EBF + 7 pp (6 weeks post‐partum); +12 pp (24 weeks post‐partum) *Association of exposures with outcomes:* IPC with EIBF and EBF (6 weeks) WhatsApp or text messages with EIBF and EBF (6 weeks) Radio spots with EBF (6 weeks) WhatsApp breastfeeding group with EBF (24 weeks)
Nigeria, 2018–2020	Flax, Ipadeola, Schnefke, Kwasa, et al. (2022)	Pre/post‐evaluation with a population‐based survey of mother‐father pairs in the same households	*Complementary feeding:* Consumption of fish +8 pp; eggs +12 pp MMF + 15 pp MAD + 11 pp *Association of exposures with outcomes:* Mothers' exposure to: Home visits from a CHEW with MDD, fish, eggs, MMF, MAD Community meetings with MDD, fish, eggs, MAD Religious services with MDD, fish, eggs, MAD TV spots with MDD, fish, eggs, MAD
Vietnam, 2010–2014	Menon, Nguyen, Saha, Khaled, Kennedy, et al. (2016)	Cluster‐randomized impact evaluation with groups assigned to intensive or nonintensive interventions	*Breastfeeding:* Feeding of prelacteals in first 3 days −19 pp Use of formula in first 3 days −22 pp EBF + 28 pp Bottle feeding −13 pp *Association of exposures with outcomes:* IPC and MM with EBF Dose response between the number of IPC exposures and EBF
	Kim et al. (2020)	Same as above	*Association of exposures with outcomes:* Exposure to both MM and IPC had additive effects on EBF
*Maternal nutrition*			
Bangladesh, 2015–2016	Nguyen et al. (2017)	Cluster‐randomized impact evaluation comparing intensive and nonintensive groups	*Maternal nutrition:* Ever use of iron‐folic acid (IFA) tablets +9.8 pp IFA tablets consumed +46 tablets Minimum dietary diversity for women (MDD‐W) + 30 pp Food groups consumed +1.6 groups Dietary intake of calcium, iron, zinc, and vitamins A, B2, folate, B12 and C increased *Breastfeeding:* No impact on EIBF EBF + 34.8 pp
Burkina Faso, 2019–2021	IFPRI (2021)	Cluster‐randomized impact evaluation comparing intensive and nonintensive groups	*Maternal nutrition:* IFA tablets consumed +21 tablets Use of 180 + IFA tablets +10.9 pp No impact on MDD‐W or adequacy of micronutrient intake. *Breastfeeding:* EIBF + 17.1 pp EBF + 8.3 pp
Ethiopia, 2019–2021	Kim et al. (2022)	Cluster‐randomized impact evaluation comparing intensive and nonintensive groups; analysis compared endline data in intervention and control groups	*Maternal nutrition:* Ever consumed IFA tablets OR 3.8 Consumed 90+ IFA tablets OR 2.3 Consumed 180+ IFA tablets OR 3.4 MDD‐W OR 1.8 *Breastfeeding:* No impact on EIBF or EBF
India, 2017–2019	Nguyen et al. (2020); Nguyen et al. (2021)	Cluster‐randomized design including cross‐sectional surveys and repeated‐measures longitudinal surveys	*Maternal nutrition:* Ever consumption of IFA tablets +9.5 pp No impact on the number of IFA consumed Ever consumption of calcium tablets +11.6 pp No impact on the number of calcium tablets consumed. No impact on MDD‐W *Breastfeeding:* No impact on EIBF EBF + 7.5 pp

Abbreviations: CM, community mobilization; EBF, exclusive breastfeeding; EIBF, early initiation of breastfeeding; IFA, iron and folic acid; IPC, interpersonal communication; MAD, minimum acceptable diet; MDD, minimum dietary diversity; MDD‐W, minimum dietary diversity for women; MM, mass media; MMF, minimum meal frequency; pp, percentage point.

A&T IYCF SBC interventions increased early initiation of breastfeeding (Bangladesh, Burkina Faso, Ethiopia), exclusive breastfeeding (Bangladesh, Burkina Faso, Ethiopia, Nigeria, Vietnam), minimum dietary diversity (Bangladesh, Ethiopia), minimum meal frequency (Bangladesh, Nigeria) and minimum acceptable diet (Bangladesh, Ethiopia, Nigeria). Associations between intervention exposures and IYCF practices were detected in Bangladesh, Ethiopia, Nigeria and Vietnam. Number of interpersonal communication exposures was associated with IYCF practices (Bangladesh, Ethiopia, Vietnam), and exposure to multiple intervention channels (e.g., interpersonal communication + community mobilization or interpersonal communication + mass media, or all three SBC channels) was more strongly associated with IYCF practices than exposure to one channel (Bangladesh, Vietnam).

A&T maternal nutrition interventions increased ever use of IFA tablets (Bangladesh, Ethiopia, India) and the number of IFA tablets consumed and/or the use of a specific number of IFA tablets (Bangladesh, Burkina Faso, Ethiopia). Maternal nutrition interventions increased minimum dietary diversity for women (Bangladesh, Ethiopia), dietary intake of micronutrients (Bangladesh), early initiation of breastfeeding (Burkina Faso) and exclusive breastfeeding (Bangladesh, Burkina Faso, India). The impact of A&T's intervention on maternal calcium tablet use was measured in India and Bangladesh only. It increased ever use of calcium tablets (India) and the number of calcium tablets consumed (Bangladesh).

### 3.3 Lessons learned from A&T SBC implementation

Key lessons learned in all or multiple A&T countries are summarized with illustrative quotations in Table 3. A full list of lessons learned, including those that were specific to one or two countries, are found in Supporting Information: Tables.

**Table 3:** Key lessons learned from A&T SBC lmplementation for IYCF and maternal nutrition.

Lessons learned	Illustrative quotations
*Interpersonal communication*	
Use and reinforce government health system (All)	‘We created here a state‐level pool for trainers on breastfeeding and complementary feeding. The pool has started to support at their district level training programme for service providers, for doctors and for staff nurses, and other frontline workers.’ India ‘We provided technical support to the government. We integrated this within the standards for the improvement of the quality of our maternal and neonatal care in health facilities.’ Burkina Faso
Improve counselling skills of health workers and community health workers/volunteers (All)	‘Capacity development of the service providers, then some sort of quality improvement activities—that is, quality training, quality counselling techniques with extended materials. They must have some sort of coaching and mentoring process and, at times, some sort of monitoring and learning process.’ Bangladesh
Deliver the messages at the appropriate time in the life cycle (All)	‘So, it's really the key message was delivered based on the age of the children.’ Vietnam ‘We have these kinds of stages, and our messages were targeted with time. So, the right message at the right time to the target group.’ Ethiopia
Provide health workers and community workers with physical SBC tools (Burkina Faso, Ethiopia, India, Nigeria, Vietnam)	‘We provided them a tool, a simple tool, field tested tool.’ Ethiopia
*Community mobilization*	
Identify and use a variety of existing platforms for community mobilization (All)	‘These influencers also continue beyond the home visits, beyond the community sensitization, through various natural groups, men's group, women's group, religious organizations, marketplace meetings and so on.’ Nigeria ‘There are two community‐based events organized in the field, in the community.… So in both the events, we provided support to the department of Integrated Child Development Services to finalize the guidelines of both the events and also supported them to have this particular event in the field, and also supported them to monitor the scene.’ India
Engage trusted leaders and influential people at different levels to share messages and get other people to participate (Bangladesh, Burkina Faso, Ethiopia, Nigeria)	‘The teachers, the religious leaders, the political leaders, they were also brought into the loop [on] why complementary feeding is good. And they were also requested [to] talk to their general community whenever they have the chance.’ Bangladesh ‘We do social mobilization through the networks of professional associations, medical associations, like a midwife, an obstetrician team, or pediatricians' association at a higher level.… We worked with a network of traditional leaders at a higher level, because they have a kind of platform at the national level, or at regional level.… We worked with networks of journalists in nutrition.’ Burkina Faso
Involve key influencers and decision makers, especially husbands and mothers‐in‐law (Bangladesh, Burkina Faso, Ethiopia, India, Nigeria, Global)	‘Pregnant women were asked to bring their husbands during the first or second visit to the health facility to get information on maternal nutrition. This was important because men hold the purse strings.’ Ethiopia ‘Bringing community mobilization in there to provide another platform, not only for mothers but now broadening the group of individuals who influence her and having the opportunity to discuss it in a group format was very important.’ Global
Be clear about the actions you want influencers to take (Bangladesh, Ethiopia, India, Global)	‘In addition to the woman, other family members need to be targeted. So, if you have multiple contacts, in those contacts you need to put different messages, overlapping messages. Not conflicting messages for different [influencers]. So, for every [influencer], you need to have a specific action. That is a good lesson that we learned. If you are going to engage husbands, you need to come up with what action the husband needs to take.’ Ethiopia
*Mass media*	
Base the media plan on an assessment of the media habits of the target population and their influencers (All)	‘At the time we started, Lagos had 111 radio stations while Kaduna had only four, so how do you deal with 111 radio stations when you have limited resources and you want to reach a specific population? So, our understanding on the audience analysis in terms of the media habits was very important.’ Nigeria
Use media professionals to create the media products (Burkina Faso, Ethiopia, Nigeria, Vietnam, Global)	‘Going for the best talent to make the change, it's super important. It's a good investment because it reaches so many people and it's durable.’ Global
Make the messages appropriate for different regions and translate them into various languages (Burkina Faso, Ethiopia, Nigeria, Vietnam)	‘We identified the difficulties and bad practices in each zone and developed messages adapted to each zone.’ Burkina Faso
Make the TV spots memorable and include relatable, realistic characters (Bangladesh, Ethiopia, Nigeria, Vietnam)	‘At that time, we could hire very famous movie stars. I don't know about movie star, but a famous person. And everyone knows about her. That makes the advertisement stand out among many other ones.’ Vietnam
Make the campaign name memorable and meaningful to all target populations (Burkina Faso, Nigeria)	‘Why did we call it Start Strong? … That came from the overall understanding of the audience, when the formative results came out one thing became very dominant and it became the base, which reflected across all populations.’ Nigeria
Create a media‐dark strategy for areas without TV or radio (Bangladesh, Ethiopia, Vietnam)	‘It's got a radio component [in areas without TV access].… They [go to] the community level and they put on the speaker. That thing got popular in the rural area.’ Vietnam
Consider frequent airing of TV or radio spots to create more awareness and cover multiple time slots, so people do not miss some of the messages or segments (Ethiopia, Nigeria, Vietnam)	‘Sometimes when you want to get something to be a popular culture, just do it over and over again. These jingles were going on in radio, from time to time. People were familiar with those radio contents that were developed.’ Nigeria
Select a small number of messages and divide them up into small doable actions (Burkina Faso, Global)	‘Identify what are the main issues, offer some kind of small, doable actions, and try to support them accordingly. And it should be step by step.’ Burkina Faso
Use talk shows or call‐in programmes to create more awareness (Bangladesh, Burkina Faso, Nigeria)	‘Another thing which the government has actually taken up from us—we call it talk shows, where experts sit around [a] table, two–three experts, and they talk to the audience through the television media or the radio, and people are allowed to ring them up and ask questions.’ Bangladesh
*SBC materials and messages*	
Use the same messages, images and branding across SBC channels for reinforcement and consistency (Ethiopia, Burkina Faso, India, Nigeria, Vietnam)	‘It is being imparted by the frontline worker, then it is given by the social mobilization, then mass communication through papers, newspaper and other television and all. So, this becomes a kind of a good approach to support the behaviour change, and this is what we have done in Bihar.’ India
Design pictorial print materials for low‐literate audiences (Burkina Faso, Ethiopia, India, Nigeria)	‘We also developed some leaflets, which were more like very low‐literate level leaflets that anybody can interpret.’ Nigeria
Contextualize visual content and language in SBC materials (Burkina Faso, Ethiopia, Nigeria, Vietnam)	‘We did specific settings and contexts for the north and the south, which made it easy for [people in] these different zones or regions to resonate and connect with the audio/visuals. They were done in local languages for better understanding and comprehension.’ Nigeria
Adapt SBC messages for specific subgroups/segment your audience (Burkina Faso, India, Nigeria, Vietnam)	‘We also understood the segmentation of our audiences. The northern part of the country has [a lot of] adolescent mothers.… We focus more on [their] significant others that would help them do the action. An adolescent mother cannot do anything without the permission of her husband, so we reached the husbands.’ Nigeria
Develop SBC tools for different users (Burkina Faso, Ethiopia, India, Nigeria, Vietnam)	‘The health workers have cards, so every time people take a certain card that's relevant to what they want to talk about, and then they explain about that.… They like it very much, because they could really plan.’ Vietnam ‘Feeding bowls motivate mothers and help them understand how much to feed their child at different ages.’ Ethiopia
Work with government and other stakeholders to create a standard set of SBC materials that are used by government and all partners (Bangladesh, Burkina Faso, India, Nigeria, Vietnam)	‘It is the Alive & Thrive intervention that made it some sort of standardized materials. Those are being used nationally. Across all the stakeholders who are actually working on IYCF. That was a big achievement.… Government has accepted those.’ Bangladesh
*Multiple SBC channels and frequent contacts*	
Multiple SBC channels create awareness, keep people engaged, and reinforce each other (All)	‘You have to have something which is constantly reminding, repeating, generating interest, mobilizing action until the next, let's say, more in‐depth, intensive interpersonal communication interaction. And then using media or community mobilization to bring other people to understand what their role is and why it's important. And what specifically about the behaviour is so important. It can't be done without other components.’ Global
Frequent contacts reinforce messages and support behaviour change (All)	‘These eight contacts permit the programme to ensure that a pregnant woman truly has all of the information clear, whether it is nutrition counselling, infant feeding, care during pregnancy or of the infant.’ Burkina Faso
*Strategic use of data*	
Collect formative data as the basis for SBC design by pinpointing problem areas related to MIYCN behaviours and identifying geographic differences (All)	‘Traditionally, before, we were targeting every aspect of breastfeeding. But what we learned from Alive & Thrive, we were able to look at the data and we were supposed to identify the gap. The problem that affects more people. A problem that has room for improvement. For example, in our Ethiopia context, [giving the baby] water is one thing that affects exclusive breastfeeding.’ Ethiopia ‘At the beginning we conducted a very big formative research to identify the enablers and barriers to IYCF behaviours. For breastfeeding, we identified that there was, I think, three challenges.’ Vietnam
Conduct a media habits assessment to design the mass media campaign (Bangladesh, Ethiopia, Nigeria, Vietnam)	‘Our understanding on the audience analysis in terms of the media habits was very important and it helped us to see, which of the media was most preferred? Which time was most appropriate? And then what location and how many times we could do that?’ Nigeria
Collaborate with government to integrate SBC indicators into regular data collection for monitoring, quality improvement and/or performance‐based incentives (Burkina Faso, Ethiopia, India, Nigeria, Vietnam)	‘The most important for me is how to hold health service providers accountable [for] breastfeeding. And the first step is to include indicators in the health system that should be tracked on [a] routine basis.’ Burkina Faso
Use data to show impact or demonstrate proof of concept (All)	‘We have conducted implementation research in Uttar Pradesh in two districts and it was [shared with] the Government of India Ministry of Health and Family Welfare department, [which convinced them] to integrate maternal nutrition in antenatal care platforms.’ India
*Systems strengthening, enabling environment and sustainability*	
Prove to the government that the model works so they can advocate for funding to integrate it into their budget (All)	‘The A&T model contributed a lot on this because it has proof of the effectiveness of an interpersonal communication model that can change the scenario, and it's the entry for us to advocate. At this moment, we are now trying to include the nutrition—we call it basic nutrition package—that will be covered by the government.’ Vietnam
Identify champion/champions for smooth programme implementation and sustainability (Bangladesh, Burkina Faso, India, Vietnam)	‘So, when the top leader is involved in some important activity, definitely it will be perpetuated at the down layer and create an enabling environment.’ India
Collaborate with and engage the government from the outset (All)	‘Engagement of the government and really proceeding with the government and just keeping them at the lead. It was a strategy that worked well. So Alive & Thrive project may be ended, but so many learnings and outcomes of Alive & Thrive will be living in the government system, national system. So that is very important, and it will continue.’ Bangladesh
Coordinate and partner with other stakeholders for sustainability, convergence of initiatives and joint advocacy (Bangladesh, Burkina Faso, India, Nigeria)	‘They made a partnership with UNICEF in terms of the mass media network component with World Bank and WHO. So, partnership was a very important strategy, actually, for what is called utilization of the resources and some sort of collaboration. And there is a complementarity and convergence of the different initiatives.’ Bangladesh
Integrate SBC programming into government platforms, support implementation, then hand over (Burkina Faso, India, Nigeria, Vietnam)	‘After the implementation research, maternal nutrition was taken up by the government as part of antenatal care and all the communication materials and training materials we developed are today the property of the Ministry of Health, which uses them for reinforcing the training of providers on interpersonal communication.’ Burkina Faso
Advocate and support the government to develop policies that create the enabling environment for the desired practices (Bangladesh, Burkina Faso, India, Nigeria, Vietnam)	‘We intensified our effort on the breast milk substitute (BMS) code which disallowed sales of breast milk substitutes in public and private facilities.… Since there was no sale of BMS, it became—let me not say ‘an incentive’ for the mothers to continue on exclusive breastfeeding—but it became obvious for the service providers to offer and support the basic option for IYCF, which is exclusive breastfeeding.’ Nigeria

Abbreviations: BMS, breast milk substitute; IYCF, infant and young child feeding; SBC, social and behavior change.

#### 3.3.1 Interpersonal communication

Participants discussed the importance of using and reinforcing government health systems to deliver interpersonal communication through training of health workers, technical support and provision of SBC tools. In all countries, A&T hired nongovernmental organizations to train and support government workers or to help reach scale by training their own community‐based cadres where government workers were not present in large enough numbers. Health worker counselling skills were flagged as a major gap and an area where A&T invested in capacity building, coaching, monitoring and training of master trainers who could continuously train new staff.

Participants also described the importance of delivering MIYCN messages to women at the appropriate times during the life cycle and making sure that health workers are trained to do so. Some participants noted that health workers tend to rattle off a list of all messages (e.g., about breastfeeding) and should be trained to prioritize a few messages and/or to adapt them to clients' needs. A global participant from the A&T Washington office explained that the impact of interpersonal communication was greatest when the health worker was able to ‘tailor the counselling to the situation of the mother and the behaviour of this child.’

#### 3.3.2 Community mobilization

Participants described the importance of identifying key influencers and getting them on board to support women in carrying out recommended MIYCN practices. This was necessary because key influencers often control the finances and make decisions that affect the purchase and intrahousehold distribution of nutritious foods. It was essential to be clear about what actions key influencers, particularly fathers/husbands and mothers/mothers‐in‐law, should take. In some contexts, nannies or grandparents needed to be included in community mobilization because they were responsible for feeding young children when mothers returned to work.

Once key influencers were known, the programme in each country could identify and use a variety of existing platforms to reach them through community mobilization activities. These ranged from men's groups to religious organizations to existing community events. It was important to engage trusted leaders, such as religious leaders, community leaders, teachers and professional associations, because people respect them and would listen to them if they shared messages. In addition, these leaders had the ability to mobilize community members to participate in maternal and child health services. In some countries, religious leaders were especially key because they are widely trusted or revered, and A&T developed talking points or sermon guides for them to use during religious services or when talking with groups.

Participants explained that support group meetings or community mobilization meetings should be led by someone from the community who is trusted, and separate groups should be held for different sets of people (i.e., mothers, men, mothers‐in‐law) so they feel comfortable raising issues of importance to them. Community mobilization group meetings were used as a platform for cooking demonstrations for complementary feeding and maternal nutrition in some countries. Demonstrations of food variety and quantity for young children and pregnant women helped them internalize the recommendations.

#### 3.3.3 Mass media

Participants said that the mass media campaign development process should be based on formative and media habits research, which is then used to identify priority behaviours and small doable actions and to develop a media buy strategy. Those initial steps should be followed by testing concepts, developing materials, pretesting and finalizing the materials, airing the spots and monitoring the campaign.

Participants described the importance of engaging media professionals to develop the mass media campaign and basing the strategy on data that could help determine which radio and TV stations to use and how frequently to air the messages. Some key lessons about messages were to make them convincing, use simple language, stick to a small number of messages linked to doable actions and adapt them for regional cultural and language differences. Some participants talked about the importance of making the campaign name memorable; making TV spots memorable by including relatable, realistic characters; and playing spots frequently to make sure people do not miss them and to create more awareness. In some countries (e.g., Vietnam and Bangladesh), famous media personalities or singers were featured in TV spots or asked to sing IYCF‐related songs for radio, which created a lot of buzz and helped people remember the messages.

Two major challenges for mass media were the cost of airing spots and reach in areas without TV or radio coverage. To address the latter issue, A&T developed media‐dark (or local media) strategies in a few countries, whereby TV and radio spots were shared in communities via projectors or battery‐powered radios with speakers during road shows, community events and group meetings. The cost of mass media remains a challenge, despite A&T's collaboration with governments in developing mass media campaigns. Participants explained that governments in most of the countries have not assigned budgets for continued airing of TV and radio spots. However, some use them for special events, such as World Breastfeeding Week.

In some countries, A&T used radio or TV talk shows or call‐in programmes as another way to create awareness and amplify mass media messages. A few also used text messaging or social media platforms, such as WhatsApp or Facebook. For example, in Nigeria, A&T and its partners created WhatsApp breastfeeding support groups led by health facility staff to motivate women to exclusively breastfeed for 6 months. Several lessons learned on mass media were specific to certain countries. For example, in Bangladesh, frontline workers were acknowledged in mass media as a way of motivating them to continue with the work of supporting families to carry out recommended MIYCN practices.

#### 3.3.4 SBC materials and messages

Participants described the importance of using the same messages, images and branding across SBC channels for consistency. Materials should include visual images, so they are easy for low‐literacy audiences to understand; be contextualized visually and linguistically; and be adapted for different target groups (e.g., mothers, fathers, mothers‐in‐law, religious leaders) or for specific users. For example, a child feeding bowl was used to support complementary feeding in Bangladesh, Ethiopia and Nigeria. It featured illustrations of diverse nutrient‐rich foods and markings indicating the quantities of food children should consume at different ages. Participants explained that messages should use simple but technically correct language; adhere to social norms; and stick to actions users should take without going into too much detail or including distracting information.

Another important lesson, which cuts across much of A&T's SBC work, was to collaborate with the government and other stakeholders in each country to create the SBC materials. Participants often described this as a long and arduous process. However, A&T's engagement with the government and stakeholders facilitated the country‐level standardization of MICYN SBC materials and the adoption of the materials for widespread use, allowing them to reach scale and build sustainability.

#### 3.3.5 Multiple SBC channels and frequent contacts

Participants across A&T countries said that multiple SBC channels (i.e., interpersonal communication, community mobilization and mass media) were important because they create awareness, keep people engaged and reinforce each other to support MIYCN practices. Participants also explained that frequent contact with mothers was needed to reinforce messages and make sure they had the support and information they needed to achieve behaviour change.

#### 3.3.6 Strategic use of data

Participants explained that data should be gathered at the beginning of the programme as the basis for SBC design. This helped identify existing MIYCN behavioural norms, pinpoint problem areas and identify geographic differences within countries. As noted in the mass media section, data were also needed to learn about media habits, develop mass media campaigns, monitor coverage of mass media activities and shift channels to increase coverage as needed. In addition, data were used to monitor SBC programme implementation, review implementation status and make changes and adaptations during implementation to improve programme quality. In several countries, this involved working with governments to integrate nutrition indicators, including indicators measuring the provision of nutrition‐related SBC, into regular health worker or community health worker data collection tools and developing processes for regularly reviewing the data. Finally, participants explained that data could be used to show impact or demonstrate proof of concept. This was an important way for A&T to have local evidence that could be used to motivate governments to integrate nutrition SBC activities into their existing systems.

#### 3.3.7 Systems strengthening, enabling environment and sustainability

A&T's work in India and its latest phases in several other countries mainly involve technical assistance and support for governments. A key lesson learned was that this approach, while slow, led to government adoption and integration of SBC processes. Across all countries, participants noted that if they could prove to the government that the SBC model worked, then they could advocate for funding within government budgets. Government budgeting for SBC was the strongest sign that the programme was sustainable.

Participants explained that it was important to collaborate with the government from the outset, integrate SBC programming into government platforms and provide support to ensure that implementation was functional. In addition, participants talked about the need to engage with other stakeholders to ensure that SBC was coordinated across partners and so all development partners could collectively collaborate with government officials. Finally, participants discussed the importance of advocacy and support of the government in developing policies that create an enabling environment for MIYCN practices and for the implementation of MIYCN SBC programmes. Relevant policies that A&T supported were related to maternity leave and the Code for the Marketing of Breast Milk Substitutes. Policies to integrate MIYCN SBC into existing government services, such as ANC, were also important because they flowed down from the national level and provided guidance for implementation at lower levels within countries.

## 4 DISCUSSION

A&T applied its SBC conceptual model across countries with adaptations or tailoring to each context based on data and collaboration with governments and their partners. None of the parts of A&T's model (i.e., interpersonal communication, community mobilization, mass media, strategic use of data and advocacy) were new, per se. A&T's innovation was designing and implementing these components of an SBC programme at scale so that they had an impact across contexts in and between countries.

A&T's approach was generally effective for increasing exclusive breastfeeding, minimum meal frequency of children and use of IFA tablets by pregnant women, but was more varied on early initiation of breastfeeding and minimum dietary diversity for children and women. Increases in early initiation of breastfeeding were attributed to A&T's IYCF SBC in Bangladesh, Burkina Faso and Ethiopia (Cresswell et al., 2019; Kim et al., 2016; Menon, Nguyen, Saha, Khaled, Sanghvi, et al., 2016), but results were mixed in Nigeria and there was no impact in Vietnam (Flax, Fagbemi, et al., 2022; Flax, Ipadeola, Schnefke, Ralph‐Opara, et al., 2022; Menon, Nguyen, Saha, Khaled, Kennedy, et al., 2016). A&T's maternal nutrition SBC had a positive impact on early initiation only in Burkina Faso (IFPRI, 2021); no effects were found in Bangladesh, Ethiopia or India (Kim et al., 2022; Nguyen et al., 2017, 2020). Early initiation of breastfeeding is influenced by many factors, including health providers' practices at delivery. Changing health workers' practices around early initiation of breastfeeding has had mixed results in different settings (Balogun et al., 2016; Martens, 2000; Tongun et al., 2019) and remains an area where further iterative or implementation research is needed. A&T had a strong impact on minimum dietary diversity for children in Bangladesh (Menon, Nguyen, Saha, Khaled, Kennedy, et al., 2016), a moderate impact in Ethiopia when an agricultural intervention was included (Kim et al., 2019), and an increase from baseline to endline was detected in Nigeria but no impact was found in difference‐in‐differences analysis (Flax, Fagbemi, et al., 2022). Increases in specific food groups in Ethiopia and Nigeria showed that A&T's SBC contributed to changes in children's diets, even if minimum dietary diversity did not shift (Flax, Ipadeola, Schnefke, Kwasa, et al., 2022; Kim et al., 2016). A&T had a strong impact on minimum dietary diversity for women in Bangladesh and the endline comparison in Ethiopia showed a difference (Kim et al., 2022; Nguyen et al., 2017), but no impact on minimum dietary diversity for women was detected in Burkina Faso or India (IFPRI, 2021; Nguyen et al., 2021). Although some A&T evaluations and other studies have shown that it is possible to increase dietary diversity in LMICs through an SBC intervention alone (Guldan et al., 2020; Roy et al., 2005; Shi et al., 2009), many factors beyond the scope of an SBC intervention remain, including household socioeconomic status, cost and availability of diverse foods, and women's empowerment and role in household decision‐making (Gatica‐Domínguez et al., 2021; Kuche et al., 2020; Shrestha et al., 2021). Most A&T interventions, except in Amhara, Ethiopia, had no associated agricultural component, which had the potential to increase the availability of a variety of foods through home production. However, even in locations with good food availability, such as the region where A&T implemented maternal nutrition SBC in Burkina Faso, impacts of the intervention on maternal dietary diversity were not seen (Kim et al., 2021), showing the complexity and ongoing challenge of using SBC to shift dietary patterns and deeply held social norms related to food consumption. It is worth noting that disruptions in food and health systems caused by COVID‐19 may confound the interpretation of dietary diversity results in countries where evaluations were ongoing during the pandemic.

The importance of interpersonal communication as a key element of A&T's intervention aligns with previous studies showing that interpersonal communication from a variety of different actors, such as peers, volunteers, community health workers and facility‐based health workers, is beneficial to support breastfeeding practices (Renfrew et al., 2012). A&T extends beyond those previous studies by demonstrating that interpersonal communication is also an essential part of SBC for complementary feeding and maternal nutrition interventions. For implementation of MIYCN SBC at scale, the present analysis shows the importance of reinforcing government facility‐based and community health systems, providing health workers and frontline workers with SBC materials, improving their counselling skills and shifting their norms about the target practices. Delivery of MIYCN messages at the appropriate time in the life cycle has long been recommended, and this concept is embedded in UNICEF's IYCF counselling messages and cards (UNICEF, 2012). This study points to the importance of training health workers and frontline workers to deliver a few messages at a time tailored to the needs of the mother and child at that moment, rather than listing all messages on a specific topic. This finding aligns with evidence that interpersonal communication in primary care works best when building on patient‐initiated discussions and through question/answer type conversations (Albury et al., 2019).

Although in many LMIC contexts women play a major role in carrying out MIYCN practices, others in their families (especially their husbands and mothers/mothers‐in‐law) and communities influence their practices (Martin et al., 2020, 2021). A&T's community mobilization strategies showed the importance of engaging with key influencers who often control decision‐making or purse strings, being clear about the actions that influencers should take, and involving trusted community and religious leaders to share messages that help shift norms. A&T used formative research to design some novel strategies for community mobilization and engagement, such as training religious leaders and providing them with sermon guides related to IYCF. This was important in Ethiopia, where the large number of fasting days limits consumption of animal source foods, and in northern Nigeria, where religious leaders are highly respected and a key way to engage men in maternal and child nutrition.

A&T's strategies for mass media and development of SBC materials were built on FHI 360's previous experience from the USAID‐funded Communication for Change project (https://www.fhi360.org/projects/communication-change-c-change) and designed using social marketing techniques (Cheng et al., 2009). Many of our findings from the interviews align with issues that are usually considered in developing a social marketing campaign—use data to assess media habits, make messages and campaign names memorable and identify small doable actions that women and key influencers can take to improve MIYCN practices. Two important additions from A&T related to media were the development of media‐dark strategies and the shift to a local media strategy, which is less expensive and could be considered for incorporation into government budgets. Sustainability of mass media investments remains a challenge because of the cost of airing TV and radio spots, and interview participants explained that governments requested A&T to develop lower‐cost media options. One important service that A&T offered countries was creating standard SBC materials by working with the government and other stakeholders to create the materials, which were then taken up by the government and all partners.

Evaluations of A&T interventions documented the association of maternal exposure to multiple SBC channels and IYCF practices, and a dose–response relationship between the number of interpersonal communication contacts and IYCF practices (Kim et al., 2020; Menon, Nguyen, Saha, Khaled, Kennedy, et al., 2016). This aligned with our participants' perceptions that multiple channels and frequent contacts with mothers and key influencers were necessary to achieve behaviour change. Participants described the need to find the sweet spot in terms of the number of interpersonal communication contacts that was adequate to share the necessary information and address MICYN challenges, but that was also feasible for mothers, health workers and frontline workers. Frequent contacts were previously shown to be beneficial for increasing some MIYCN practices, especially breastfeeding (Lamstein et al., 2014). Participants also emphasized the importance of using different SBC channels for reinforcing and reminding mothers, key influencers and other stakeholders about MIYCN messages.

Strategic use of data was cited throughout the A&T interviews. A&T used formative data for designing and pre‐testing the SBC intervention strategy, SBC materials and mass media; collected monitoring data, which was used for programme adaptation; and conducted evaluations and implementation research in collaboration with external researchers to gather evidence of impact. A&T's collection and use of formative data was built on previous research showing the importance of identifying barriers and enablers to optimal nutrition practices in designing SBC interventions (Bentley et al., 2014; Fabrizio et al., 2014; Tumilowicz et al., 2016). The initiative's collection of impact data was important for showing proof of concept for the effectiveness of SBC implementation at scale in phase 1 and was used by A&T as a tool for advocacy with governments.

This study had several strengths and limitations. Using one researcher to conduct all the interviews and the analysis has strengths in terms of consistency in using the question guide and eliminating the need for interrater reliability during coding. To ensure that other perspectives were included in the analysis, the other coauthors had an opportunity to provide input on the preliminary coding and findings. In each country, only a handful of A&T staff were involved in or knowledgeable about the technical aspects of SBC implementation. We included all of these staff in KIIs, which resulted in good data saturation in each country. This study offers a narrative review of the impact of A&T's SBC on MIYCN practices, but does not include a meta‐analysis, which would provide more definitive findings on A&T's overall intervention effects on outcomes.

## 5 CONCLUSION

In conclusion, the A&T initiative demonstrated strategies and best practices for implementing MIYCN at scale. The initiative has documented elements of its implementation strategies on its website, but this paper addresses the need for nutrition programmes not only to show effectiveness but to describe implementation strategies, successes and challenges (Menon et al., 2014). Taking into consideration lessons learned, governments and their implementing partners should replicate the A&T SBC model in different countries and contexts, document further adaptations to the A&T model, and share them widely. For nongovernmental implementers, the strategy A&T has used in India—focused on technical assistance and support to the government—lends itself to implementation at scale and is sustainable because activities are embedded in government systems rather than being project‐ or NGO‐based, which are dependent on external funding and therefore generally time limited. Though the model in India was less intense, it varied in impact (Nguyen et al., 2021) compared to the more intense intervention implemented by BRAC in Bangladesh (Menon, Nguyen, Saha, Khaled, Kennedy, et al., 2016), suggesting that examining the extent to which the India model advances sustainability deserves further research. MIYCN programmes should work to fill remaining gaps in knowledge by developing and testing implementation approaches that can consistently improve early initiation of breastfeeding and maternal and child dietary diversity at scale and by designing media that can cost‐effectively and sustainably engage people over time.

## AUTHOR CONTRIBUTIONS

Valerie L. Flax designed and performed the research, analysed the data and drafted the paper. Sujata Bose, Jessica Escobar‐DeMarco and Edward A. Frongillo conceptualized the research, provided input on the design and contributed to the manuscript.

## CONFLICT OF INTEREST STATEMENT

The authors declare no conflict of interest.

## ETHICS STATEMENT

FHI 360 institutional review board (IRB) determined that the study was not human subjects research. Ethical approval was obtained from the University of Dhaka's Institute of Health Economics IRB in Bangladesh and the IRB of the Institut de Recherche en Sciences de la Santé in Burkina Faso. The other countries did not require IRB approval because the study was part of a programme and was not considered human subjects research. For Bangladesh and Burkina Faso, signed consent forms were obtained. Although not required, for participants from other countries and the A&T Washington and regional offices, verbal assent to participate and be recorded was obtained.

## Supporting information

Supporting information.

## Data Availability

The data that support the findings of this study are available on request from the corresponding author. The data are not publicly available due to privacy or ethical restrictions.
