# Supplementary material for: Changing maternal, infant and young child nutrition practices through social and behaviour change interventions implemented at scale: Lessons learned from Alive & Thrive
Source: Matern Child Nutr. 2023 Sep 21;21(2):e13559. doi: 10.1111/mcn.13559 (PMC11956063; doi:10.1111/mcn.13559)
Supplement: Supplementary file 1 — Supporting information. [file MCN-21-e13559-s001.docx]

Supplemental Table 1. Lessons Learned on Interpersonal Communication

| Lessons learned | Illustrative quotations |
| --- | --- |
| Use and reinforce government health system (All) | “We created here a state-level pool for trainers on breastfeeding and complementary feeding. The pool has started to support at their district level training program for service providers, for doctors and for staff nurses, and other frontline workers.” India  “We provided technical support to the government. We integrated this within the standards for the improvement of the quality of our maternal and neonatal care in health facilities.” Burkina Faso |
| Improve counseling skills of health workers and community health workers/volunteers (All) | “Capacity development of the service providers, then some sort of quality improvement activities—that is, quality training, quality counseling techniques with extended materials. They must have some sort of coaching and mentoring process and, at times, some sort of monitoring and learning process.” Bangladesh |
| Deliver the messages at the appropriate time in the life cycle (All) | “So, it’s really the key message was delivered based on the age of the children.” Vietnam  “We have these kind of stages and our message was targeted with time. So, right message at the right time to the target group.” Ethiopia |
| Provide health workers and community workers with physical SBC tools (Burkina Faso, Ethiopia, India, Nigeria, Vietnam) | “We provided them a tool, a simple tool, field tested tool.” Ethiopia |
| Use a combination of one-on-one and group counseling (Burkina Faso, Nigeria) | “Usually at health facilities they will do a *causerie* with all the women who are there for ANC. Then when the women go in to see the midwife, there is no individual counseling. Through advocacy, supervision, support, we have convinced providers to do individual counseling.” Burkina Faso |
| Make sure community volunteers and fee-based health workers are incentivized so they are motivated to do the job (Burkina Faso, Ethiopia, Vietnam) | “We ensure that the providers are sufficiently rewarded and have the attitudes, beliefs, and norms that facilitate the quality breastfeeding support.” Vietnam |
| Develop master trainers for health staff (India, Nigeria) | “What we did was to create cluster master trainers, train them within the facility so that almost every facility has a master trainer who is somehow permanent in that facility. So even if they have transferred some staff, training will continue, and that’s how we did it to institutionalize our IPC system.” Nigeria |
| Convince frontline workers about complementary feeding practices (India, Global) | “Our frontline workers were horrified that we could ever imagine that this little baby of seven months old would be eating so much food and solid food and so many times a day.… So breaking down the barriers in the minds of the people who are supposed to carry out IPC for complementary feeding is a huge barrier. So, they need to be brought on board in a very deliberate and careful manner.” Global |
| Allow mothers with successful experiences to share information during group counseling (Burkina Faso, Nigeria) | “They were able to share experiences among themselves. They’re able to use themselves as examples.… You find where a caregiver can come out and confidently say that she did exclusive breastfeeding and it worked for her. And she’s holding the evidence, which is a healthy child, and other women, pregnant women or lactating women, mothers are actually learning from it.” Nigeria |
| Prioritize a few messages instead of providing a whole list (Burkina Faso, Global) | “Not going for all the messages in one shot, but prioritize one or two things.” Global |
| Layer nutrition interpersonal communication onto other programs (India) | “We have here … the Social Mobilization Network. The network was built up for the polio eradication program.… We have layered the MIYCN intervention with the polio messages.” India |

Supplemental Table 2. Lessons Learned on Community Mobilization

| Lessons learned | Illustrative quotations |
| --- | --- |
| Identify and use a variety of existing platforms for community mobilization (All) | “These influencers also continue beyond the home visits, beyond the community sensitization, through various natural groups, men’s group, women’s group, religious organizations, marketplace meetings and so on.” Nigeria  “There are two community-based events organized in the field, in the community.… So in both the events, we provided support to the department of ICDS to finalize the guidelines of both the events and also supported them to have this particular event in the field, and also supported them to monitor the scene.” India |
| Engage trusted leaders and influential people at different levels to share messages and get other people to participate (Bangladesh, Burkina Faso, Ethiopia, Nigeria) | “The teachers, the religious leaders, the political leaders, they were also brought into the loop [on] why complementary feeding is good. And they were also requested [to] talk to their general community whenever they have the chance.” Bangladesh  “We do social mobilization through the networks of professional associations, medical associations, like a midwife, an obstetrician team, or pediatricians association at a higher level.… We worked with a network of traditional leaders at a higher level, because they have a kind of platform at the national level, or at regional level.… We worked with networks of journalists in nutrition.” Burkina Faso |
| Involve key influencers and decision makers, especially husbands and mothers-in-law (Bangladesh, Burkina Faso, Ethiopia, India, Nigeria, Global) | “Pregnant women were asked to bring their husbands during the first or second visit to the health facility to get information on maternal nutrition. This was important because men hold the purse strings.” Ethiopia  “Bringing community mobilization in there to provide another platform, not only for mothers but now broadening the group of individuals who influence her and having the opportunity to discuss it in a group format was very important.” Global |
| Be clear about the actions you want influencers to take (Bangladesh, Ethiopia, India, Global) | “In addition to the woman, other family members need to be targeted. So, if you have multiple contacts, in those contacts you need to put different messages, overlapping messages. Not conflicting messages for different [influencers]. So, for every [influencer], you need to have a specific action. That is a good lesson that we learned. If you are going to engage husbands, you need to come up with what action the husband needs to take.” Ethiopia |
| Design support group or community meetings so they are led by a person the community trusts and who is from the community (Nigeria, Vietnam) | “The ones who facilitated the meetings are local people, so they could speak the local language, and they’re the ones who can be trusted by other people in the community. So that makes the meetings very good. The facilitators tend to be very confident about what they’re doing, and then they could have the connection with other people, and then the mothers who attend the meeting can very freely talk about their problems.” Vietnam |
| Design support group or community activities with separate groups for different people, so they feel comfortable having a discussion (Nigeria, Vietnam) | “We try to not mix groups. Because we really want to hear from people, we don’t want to have someone who is in a different group. A mother-in-law sitting with the pregnant woman, and maybe the mother themself doesn’t want to speak because they are afraid to offend the older lady.” Vietnam |
| Include cooking demonstrations in community mobilization for complementary feeding and maternal nutrition (Bangladesh, Ethiopia, India, Nigeria, Global) | “In complementary feeding, the key issue that needed to be emphasized was it required a demonstration. That demonstration was for the caregivers, mothers to understand what exactly complementary feeding means.” Nigeria |
| Involve grandparents, nannies, or daycare workers in community mobilization in contexts where mothers go back to work (Nigeria, Vietnam) | “In one area, many times we worked very hard but then we had to get the nanny because they are the one who cooks for the children, and many times they are the one who come from the rural area and don’t have any skill.” Vietnam |
| Understand local context to identify right influencers and then test and adapt the strategy (Bangladesh, Nigeria, Global) | “Social mobilization will depend on the local context because identification of the local influentials varies from society to society. It varies from the rural area or urban, different partners are there. So, it is a local level context.” Bangladesh |
| Give participants assignments to do after community mobilization activities to provide reinforcement through community acknowledgement (Ethiopia) | “If we give them one assignment to practice, [it] will empower them and make them [able to] bring a change in the community. Unless we do that, if we focus only to give them information [or] knowledge, that cannot change.” Ethiopia |

Supplemental Table 3. Lessons Learned on Mass Media

| Lessons learned | Illustrative quotations |
| --- | --- |
| Base the media plan on an assessment of the media habits of the target population and their influencers (Bangladesh, Burkina Faso, Ethiopia, Nigeria, Vietnam, Global) | “At the time we started Lagos had 111 radio stations while Kaduna had only four, so how do you deal with 111 radio stations when you have limited resources, and you want to reach a specific population? So, our understanding on the audience analysis in terms of the media habits was very important.” Nigeria |
| Use media professionals to create the media products (Burkina Faso, Ethiopia, Nigeria, Vietnam, Global) | “Going for the best talent to make the change, it’s super important. It’s a good investment because it reaches so many people and it’s durable.” Global |
| Make the messages appropriate for different regions and translate into various languages (Burkina Faso, Ethiopia, Nigeria, Vietnam) | “We identified the difficulties and bad practices in each zone and developed messages adapted to each zone.” Burkina Faso |
| Make the TV spots memorable and include relatable, realistic characters (Bangladesh, Ethiopia, Nigeria, Vietnam) | “At that time, we could hire very famous movie stars. I don’t know about movie star, but a famous person. And everyone knows about her. That makes the advertisement stand out among many other ones.” Vietnam |
| Make the campaign name memorable and meaningful to all target populations (Burkina Faso, Nigeria) | “Why did we call it Start Strong? … That came from the overall understanding of the audience, when the formative results came out one thing became very dominant and it became the base, which reflected across all populations.” Nigeria |
| Create a media-dark strategy for areas without TV or radio (Bangladesh, Ethiopia, Vietnam) | “It’s got a radio component [in areas without TV access].… They [go to] the community level and they put on the speaker. That thing got popular in the rural area.” Vietnam |
| Consider frequent airing of TV or radio spots to create more awareness and cover multiple time slots, so people do not miss some of the messages or segments (Ethiopia, Nigeria, Vietnam) | “Sometimes when you want to get something to be a popular culture, just do it over and over again. These jingles were going on in radio, from time to time. People were familiar with those radio contents that were developed.” Nigeria |
| Select a small number of messages and divide them up into small doable actions (Burkina Faso, Global) | “Identify what are the main issues, offer some kind of small, doable actions, and try to support them accordingly. And it should be step by step.” Burkina Faso |
| Use talk shows or call-in programs to create more awareness (Bangladesh, Burkina Faso, Nigeria) | “Another thing which the government has actually taken up from us—we call it talk shows, where experts sit around [a] table, two–three experts, and they talk to the audience through the television media or the radio, and people are allowed to ring them up and ask questions.” Bangladesh |
| Use social media platforms (Bangladesh, Burkina Faso, Nigeria) | “I think one aspect that can be fully explored is the social media, taking advantage of social media platform because various people of different age categories of different generations who have access to mobile phones and equally would get data, they have such platforms.” Nigeria |
| Find the angle that will convince or appeal to people (Bangladesh, Nigeria) | “We told them your child will be brainy.…These things, catchy words, which the local people would like, it has to appeal to them.… If you just tell them, oh, eat egg, eat chicken, it doesn’t matter that much; but once you talk about the benefit, it matters.” Bangladesh |
| Use simple language (Bangladesh, Nigeria) | “In breastfeeding we always communicate too technical. We need to simplify the technicality in the language of the communication so that they will understand.” Nigeria |
| Use mass media to create awareness and shift social norms and get the whole family behind the appropriate norms (Bangladesh) | “In most countries when you see a television the whole family sits together. So, the whole family actually watches that, okay, this good for the child and we have a child in our house. So, then the whole family’s convinced together.” Bangladesh |
| Use mass media to remind frontline workers of key messages as a way of motivating them (Bangladesh) | “We found in Bangladesh that the frontline workers said they would’ve given up on complementary feeding if it hadn’t been for mass media because they were reminded constantly through mass media as to what are the key messages to focus on.” Global |
| Use mass media to provoke interpersonal communication (Bangladesh, Global) | “If you do something in a way that people want to talk about it, that gets you to having generated interpersonal communication on your topic on breastfeeding, through mass media, and then the synergistic effect of the media messaging itself. Plus, that interaction that happened is far more powerful.” Global |
| Use mass media to convince key influencers during community mobilization sessions (Bangladesh) | “We would go from one union to another union, and we would show the TV commercials there. So, the political leaders, they would see it and they would all say that I’m committed now, I will personally go from house to house.” Bangladesh |
| Acknowledge volunteers and frontline workers through mass media to motivate them (Ethiopia) | “There was a radio program, a radio drama, short drama that was airing in the national as well as regional radios. So, in that drama there was a place that recognized the role of community volunteers who are doing those activities.” Ethiopia |
| Reserve enough time to go through all of the steps of designing a media campaign, including formative research, design, pretesting, etc. (Vietnam) | “In order to have such campaign launch, it takes a lot of time because we have to do the formative research. We have to design, we have to follow all the steps, pretesting and so on.” Vietnam |
| Form alliances with emerging social media groups to extend reach of mass media messages (Vietnam) | “When we see that there are emerging and influential groups in society, we map them out, and have the discussion with them, and influence them in the way that we provide and feed them with knowledge, with information, updated information. And we became a kind of alliance.” Vietnam |
| Run mini campaigns to address specific issues (Bangladesh) | “[In Bangladesh], we had to run a whole mini campaign on hand washing as part of complementary feeding. Not as part in one campaign, but a separate mini mass media campaign.” Global |
| Support the government on mass media messages and communication strategies (India) | “At that time, we were supporting GHS for the mass media activity to create the messages, to create the technical document, to create the advocacy document for breastfeeding, complementary feeding this kind of activity for the mass media publication. So, our role was to support the partners, our strategy partner.” India |
| Use projectors in health facilities to share TV spots in waiting areas (Nigeria) | “With or without light, those projectors were being used to project nutrition messages during antenatal care, during child welfare, and other programs in the facility. If the battery is low, they charge it.” Nigeria |

Supplemental Table 4. Lessons Learned on SBC Materials and Messages

| Lessons learned | Illustrative quotations |
| --- | --- |
| Use the same messages, images, and branding across SBC channels for reinforcement and consistency (Ethiopia, Burkina Faso, India, Nigeria, Vietnam) | “It is being imparted by the frontline worker, then it is given by the social mobilization, then mass communication through papers, newspaper, and other television and all. So, this becomes a kind of a good approach to support the behavior change, and this is what we have done in Bihar.” India |
| Design pictorial print materials for low literate audiences (Burkina Faso, Ethiopia, India, Nigeria) | “We also developed some leaflets, which were more like very low-literate level leaflets that anybody can interpret.” Nigeria |
| Contextualize visual content and language in SBC materials (Burkina Faso, Ethiopia, Nigeria, Vietnam) | “We did specific settings and contexts for the north and the south, which made it easy for [people in] these different zones or regions to resonate and connect with the audio/visuals. They were done in local languages for better understanding and comprehension.” Nigeria |
| Adapt SBC messages for specific subgroups/segment your audience (Burkina Faso, India, Nigeria, Vietnam) | “We also understood the segmentation of our audiences. The Northern part of the country has [a lot of] adolescent mothers.… We focus more on [their] significant others that would help them do the action. An adolescent mother cannot do anything without the permission of her husband, so we reached the husbands.” Nigeria |
| Use simple but technically correct language in messages and materials (Burkina Faso, India, Nigeria) | “Technical support was there—like getting the proper messages, giving the right kind of a communication—that kind of a support we have provided for the mass media.” India |
| Develop SBC tools for different users (e.g., counseling cards for health workers, feeding bowls for mothers to use with children) (Burkina Faso, Ethiopia, India, Nigeria, Vietnam) | “The health workers have cards, so every time people take a certain card that’s relevant to what they want to talk about, and then they explain about that.… They like it very much, because they could really plan.” Vietnam  “Feeding bowls motivate mothers and help them understand how much to feed their child at different ages.” Ethiopia |
| Involve influencers (e.g., religious leaders, newspaper editors) in SBC by providing them with talking points (Bangladesh, Nigeria) | “The editor of a newspaper, he said, ‘Okay, you give me a sort of an advertisement, which I’ll put in my newspaper and I’ll promote it myself.’” Bangladesh |
| Work with government and other stakeholders to create a standard set of SBC materials that are used by government and all partners (Bangladesh, Burkina Faso, India, Nigeria, Vietnam) | “It is the Alive & Thrive intervention that made it some sort of standardized materials. Those are being used nationally. Across all the stakeholders who are actually working on IYCF. That was a big achievement.… Government has accepted those.” Bangladesh |
| Understand and make use of social norms to craft SBC messages (Burkina Faso, India, Nigeria) | “So, understanding the culture, understanding what to communicate in such a way that people will be able to see the benefit without necessarily being offended by assuming that their cultural practices were wrong.” Nigeria |
| Keep the message to the action you want people to take and do not include distractions (Nigeria) | “We shouldn’t waste time on making that emphasis that creates suspicion. We should emphasize that the breast milk contains water that the baby needs and that’s why it looks that voluminous in terms of quantity of the liquid.” Nigeria |
| Keep adapting SBC to the time and situation (Vietnam) | “So, I think the project, especially SBC, it’s very dependent on the time of implementation. Maybe a few years ago, it’s very correct, but if we apply the same thing at this point, it’s not appropriate.… For example, a few years ago with A&T, we were using paper, we were using direct communication. But at this point, if we apply the same thing, maybe it’s not very effective because now they use more social media, using apps, for example, Facebook or Twitter.” Vietnam |

Supplemental Table 5. Lessons Learned Related to Multiple SBC Channels and Frequent Contacts

| Lessons learned | Illustrative quotations |
| --- | --- |
| Multiple SBC channels create awareness, keep people engaged, and reinforce each other (All) | “You have to have something which is constantly reminding, repeating, generating interest, mobilizing action until the next, let's say, more in-depth, intensive interpersonal communication interaction. And then using media or community mobilization to bring other people to understand what their role is and why it’s important. And what specifically about the behavior is so important. It can’t be done without other components.” Global |
| Frequent contacts reinforce messages and support behavior change (All) | “These eight contacts permit the program to ensure that a pregnant woman truly has all of the information clear, whether it is nutrition counseling, infant feeding, care during pregnancy or of the infant.” Burkina Faso |

Supplemental Table 6. Lessons Learned Related to Use of Data

| Lessons learned | Illustrative quotations |
| --- | --- |
| Collect formative data as the basis for SBC design by pinpointing problem areas related to MIYCN behaviors and identifying geographic differences (All) | “Traditionally, before we were targeting every aspect of breastfeeding. But what we learned from Alive & Thrive, we were able to look at the data and we were supposed to identify the gap. The problem that affects more people. A problem that has room for improvement. For example, in our Ethiopia context, [giving the baby] water is one thing that affects exclusive breastfeeding.” Ethiopia  “At the beginning we conducted a very big formative research to identify the enablers and barriers to IYCF behaviors. For breastfeeding, we identified that there was, I think, three challenges.” Vietnam |
| Conduct a media assessment to design the mass media campaign (Bangladesh, Ethiopia, Nigeria, Vietnam) | “Our understanding on the audience analysis in terms of the media habits was very important and it helped us to see, which of the media was most preferred? Which time was most appropriate? And then what location and how many times we could do that?” Nigeria |
| Collaborate with government to integrate SBC indicators into regular data collection for monitoring, quality improvement, and/or performance-based incentives (Burkina Faso, Ethiopia, India, Nigeria, Vietnam) | “The most important for me is how to hold health service providers accountable [for] breastfeeding. And the first step is to include indicators in the health system that should be tracked on [a] routine basis.” Burkina Faso |
| Use data to show impact or demonstrate proof of concept (All) | “We have conducted implementation research in Uttar Pradesh in two districts and it was [shared with] the Government of India Ministry of Health and Family Welfare department, [which convinced them] to integrate maternal nutrition in antenatal care platforms.” India |
| Give health providers checklists and performance indicators related to MIYCN SBC (Vietnam) | “We ensure that we have clear performance indicators and checklists so that the health staff, the health providers can know what they are expected to do.” Vietnam |

Supplemental Table 7. Lessons Learned Related to Systems Strengthening, Enabling Environment, and Sustainability

| Lessons learned | Illustrative quotations |
| --- | --- |
| Prove to government that the model works so they can advocate for funding to integrate it into their budget (All) | “The A&T model contributed a lot on this because it has proof of the effectiveness of an interpersonal communication model that can change the scenario, and it’s the entry for us to advocate. At this moment, we are now trying to include the nutrition—we call it basic nutrition package—that will be covered by the government.” Vietnam |
| Convince government to incorporate costs related to nutrition SBC into annual budgets (Burkina Faso, India) | “This time, collectively, we have advocated with the government to incorporate maternal nutrition activity in the annual PIP, project implementation plan.” India |
| Identify champion/champions for smooth program implementation and sustainability (Bangladesh, Burkina Faso, India, Vietnam) | “So, when the top leader is involved in some important activity, definitely it will be perpetuated at the down layer and create an enabling environment.” India |
| Collaborate with and engage government from the outset (All) | “Engagement of the government and really proceeding with the government and just keeping them at the lead. It was a strategy that worked well. So Alive & Thrive project maybe ended, but so many learnings and outcomes of Alive & Thrive will be living in the government system, national system. So that is very important, and it will continue.” Bangladesh |
| Coordinate and partner with other stakeholders for sustainability, convergence of initiatives, and joint advocacy (Bangladesh, Burkina Faso, India, Nigeria | “They made a partnership with UNICEF in terms of the mass media network component with World Bank and WHO. So, partnership was a very important strategy, actually, for what is called utilization of the resources and some sort of collaboration. And there is a complementarity and convergence of the different initiatives.” Bangladesh |
| Integrate SBC programming into government platforms, support implementation, then hand over (Burkina Faso, India, Nigeria, Vietnam) | “After the implementation research, maternal nutrition was taken up by the government as part of antenatal care and all the communication materials and training materials we developed are today the property of the Ministry of Health, which uses them for reinforcing the training of providers on interpersonal communication.” Burkina Faso |
| Advocate and support government to develop policies that create the enabling environment for the desired practices (Bangladesh, Burkina Faso, India, Nigeria, Vietnam) | “We intensified our effort on the breast milk substitute (BMS) code which disallowed sales of breast milk substitutes in public and private facilities.… Since there was no sale of BMS, it became—let me not say ‘an incentive’ for the mothers to continue on exclusive breastfeeding—but it became obvious for the service providers to offer and support the basic option for IYCF, which is exclusive breastfeeding.” Nigeria  “We need to create enabling environment at the policy level. The policies should be in place in the state so that the officials follow the guidelines to implement the program through their workforce. So, the policies should be there, and the enabling environment should be there. For the enabling environment, we have all the technical expertise, as well as tools and materials should be there in place so that they can use them, and they can train their service providers on that.” India |
